# Development of a Novel Modified High-Modulus Asphalt Based on Waste Rubber Powder

**DOI:** 10.3390/ma18184314

**Published:** 2025-09-15

**Authors:** Maowen Li, Chao Pu, Ping Zheng, Waiti Litifu, Zhe Ma, Peng Yin

**Affiliations:** 1 Xinjiang Transportation Investment (Group) Co., Ltd., Urumqi 830011, China; 2 Xinjiang Key Laboratory for Safety and Health of Transportation Infrastructure in Alpine and High-Altitude Mountainous Areas, Urumqi 830011, China; 3 Xinjiang Transport Planning Survey and Design Institute Co., Ltd., Urumqi 830011, China; 4 School of Infrastructure Engineering, Dalian University of Technology, No. 2, Linggong Road, Ganjingzi District, Dalian 116024, China; 5 Xinjiang Communications Investment Construction Management Co., Ltd., Urumqi 830011, China

**Keywords:** modified asphalt, orthogonal tests, rheological properties, mix proportion, modification mechanism

## Abstract

This study developed a novel waste rubber powder modified high-modulus asphalt based on composite modification technology. The preparation process was determined by orthogonal tests, and the mechanical properties of modified high-modulus asphalt were evaluated through rheological tests in comparison with three common high-modulus asphalts to verify its application potential. The modification mechanism of modified high-modulus asphalt was characterized by Fourier transform infrared spectroscopy and fluorescence microscopy tests. The results show that the recommended mixing ratio of modified high-modulus asphalt is 20% waste rubber, 6% ethylene-vinyl acetate copolymer, and 4% ammonium polyphosphate. Although the high temperature performance, rutting resistance, and fatigue performance of modified high-modulus asphalt are slightly inferior to those of high-modulus asphalts prepared with two polymer modifiers, they are significantly better than those of hard asphalt, and all mechanical properties meet the application requirements of high-modulus asphalt. Compared with other asphalts, modified high-modulus asphalt exhibits the most prominent low temperature performance. The microscopic test results indicate that the modification process of modified high-modulus asphalt is chemical modification with minimal swelling, and no obvious network structure is formed inside.

## 1. Introduction

With the continuous acceleration of urbanization and the widespread popularization of transportation tools in recent years, road construction and maintenance have become increasingly important issues of social concern. Traditional asphalt mixtures have certain performance limitations in coping with working conditions such as traffic loads and temperature fluctuations, urgently requiring research and optimization of material properties to enhance the strength, stability, and durability of road structures [1,2,3]. As a new type of road material, high-modulus asphalt can effectively resist the dynamic stress caused by tire loads and temperature changes, relying on its high elastic modulus and deformation resistance [4]. Given its excellent high temperature performance and fatigue resistance, high-modulus asphalt has gradually become a key material for constructing high temperature and heavy-load road sections [5].

The difference between high-modulus asphalt and base asphalt or ordinary-modified asphalt lies in its higher complex shear modulus (G*). Relevant studies have shown that G* is a key index distinguishing high-modulus asphalt from other asphalts, with the requirement that G* (60 °C, 10 Hz, 12%) ≥ 10 kPa [6]. Current preparation methods for high-modulus asphalt mainly include using low-grade hard asphalt, high-modulus modifiers, and high-dosage polymer modifiers [7]. For example, Khiavi [8] evaluated the feasibility of using two types of hard asphalt to develop high-modulus asphalt and found that the mechanical properties of high-modulus asphalt mixtures prepared with hard asphalt were significantly improved compared with traditional asphalt. Zou [9] used high-modulus modifiers to prepare high-modulus modified asphalt mixtures and compared them with ordinary asphalt mixtures, revealing that the high temperature performance, water stability, and dynamic modulus of the modified mixtures were significantly enhanced. Wang [10] prepared high-modulus modified asphalt with high-modulus modifiers and compared it with styrene-butadiene-styrene (SBS) modified asphalt; rheological test results showed that the rutting resistance of the modified asphalt prepared with high-modulus modifiers exceeded that of SBS modified asphalt. Mi [11] used a composite high-modulus modifier to prepare high-modulus asphalt mixtures and analyzed their road performance through mechanical tests, finding that although the high temperature performance of the mixtures increased with the dosage of the high-modulus modifier, their low temperature cracking resistance significantly decreased. Wu [12] used high-modulus modifiers to prepare high-modulus modified asphalt mixtures and analyzed their cracking resistance under different aging conditions, demonstrating that aging weakens the cracking resistance of asphalt mixtures, while the incorporation of high-modulus modifiers can reduce the impact of aging to a certain extent. Li [13] prepared high-modulus asphalt using SBS modifiers and rock asphalt, showing that their modification effects could effectively enhance the high temperature performance of base asphalt and reduce its temperature sensitivity. In summary, the current preparation of high-modulus asphalt mainly relies on hard asphalt, high-modulus modifiers, and high-dosage polymers, which largely restricts the development of high-modulus asphalt [14,15]. Specifically, hard asphalt has poor low temperature performance, easily leading to a decline in the service performance of asphalt pavements; high-modulus modifiers and high-dosage polymer modifiers have problems such as large dosage and high cost in application, making them difficult to meet the development needs of green and economical asphalt pavements, thus severely limiting the promotion and application of high-modulus asphalt.

The relevant studies have shown that the WR can undergo physical and chemical interactions with asphalt molecules, enhancing their adhesion and strength by improving asphalt viscosity and polymerizability [16]. Meanwhile, WR can fill the micro-pores inside asphalt, improving compactness and stability, and further optimizing the fatigue resistance and aging resistance of asphalt [17,18]. This characteristic endows WR with application potential in the development of high-modulus asphalt. In addition, the continuous maturity of modified asphalt technology provides technical support for the research and development of WR-based high-modulus asphalt. However, it is worth noting that an excessively high content of WR can easily lead to the degradation of asphalt’s low temperature performance. Therefore, in practical applications, the content of WR in asphalt is often limited to 15%. Therefore, based on the theory of composite materials, this study developed a high-modulus asphalt using WR as the main material through orthogonal tests, with the aim of further increasing the utilization rate of WR while reducing its adverse impact on the low temperature performance of asphalt. By comparing it with three common high-modulus asphalts and combining physical tests, rheological tests, and Fourier transform infrared spectroscopy (FTIR) analysis, the performance characteristics and modification mechanism of WR modified high-modulus asphalt were systematically explored, aiming to provide a reference for the engineering application of high-modulus asphalt.

## 2. Materials and Methods

### 2.1. Materials

The virgin asphalt used was 70# base asphalt (VA). The materials for developing high-modulus modified asphalt included waste rubber powder (WR) from Baixin New Materials Technology Co., Ltd., Foshan, China; ammonium polyphosphate (PPA) from Yousuo Chemical Technology Co., Ltd., Linyi, China; and ethylene-vinyl acetate copolymer (EVA) from Xinqicheng Plasticizing Co., Ltd., Suzhou, China. The other three types of high-modulus modified asphalts were high-modulus asphalt prepared with 35# hard asphalt (HGA), high-modulus agent modified asphalt (HMA), and high-dosage SBS modified asphalt (SBA). The properties of the asphalts and modified materials involved in the study are shown in Table 1 and Table 2.

### 2.2. Methods

#### 2.2.1. Orthogonal Test

To address the performance limitations of existing high-modulus asphalts, this study selected WR, EVA, and PPA to prepare modified asphalt. Previous research has shown that the rubber components in WR can enhance the bonding strength of asphalt and improve its compactness and stability by filling micro-pores; EVAs can chemically react with asphalt to improve its flexural toughness and crack resistance; PPA increases asphalt viscosity and elastic modulus through hydrogen bonding. These material characteristics provide a feasible basis for developing modified high-modulus asphalt. Therefore, an orthogonal test was designed to optimize the mixing formula. According to relevant studies [19,20], the dosage ranges were set as follows: 10~20% for WR, 4~6% for EVA, and 2~4% for PPA. The specific factor levels are presented in Table 3.

#### 2.2.2. Preparation Process of Modified High-Modulus Asphalt

To meet the requirement of G* (60 °C, 10 Hz, 12%) ≥ 10 kPa for high-modulus asphalt specimens, the preparation process of waste rubber powder modified high-modulus asphalt (WRA) was determined through extensive preliminary tests as follows: First, VA was heated in an oven at 150 °C until it became flowable. Then, a certain amount of WR was added, and the mixture was sheared at 5000 r/min for 0.5 h using a high-speed shearing machine from Xinle Electromechanical Technology Co., Ltd. in Shanghai, China. Next, PPA and EVA were sequentially added according to the formula determined by the orthogonal test, and shearing was continued at the same speed for 0.5 h. After shearing, the specimen was cured in an oven at 150 °C for 0.5 h to obtain WRA.

#### 2.2.3. Physical Property Tests

To characterize the effects of different modifiers on the physical properties of asphalt, penetration, ductility, and softening point tests were conducted on the modified high-modulus asphalt according to the specifications of JTG E20-2011 [21].

#### 2.2.4. Rheological Test Methods

To evaluate the comprehensive service performance of the self-developed modified high-modulus asphalt, a temperature sweep test, multiple stress creep recovery (MSCR) test, linear amplitude sweep (LAS) test, and bending beam rheometer (BBR) test were performed to assess the high temperature performance, rutting resistance, fatigue resistance, and low temperature cracking resistance of the asphalt samples.

**Figure 1:**
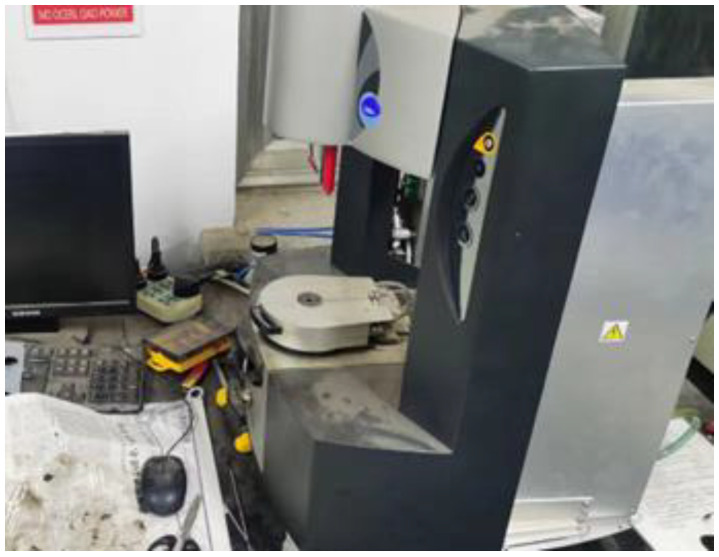
The dynamic shear rheometer.

**Figure 2:**
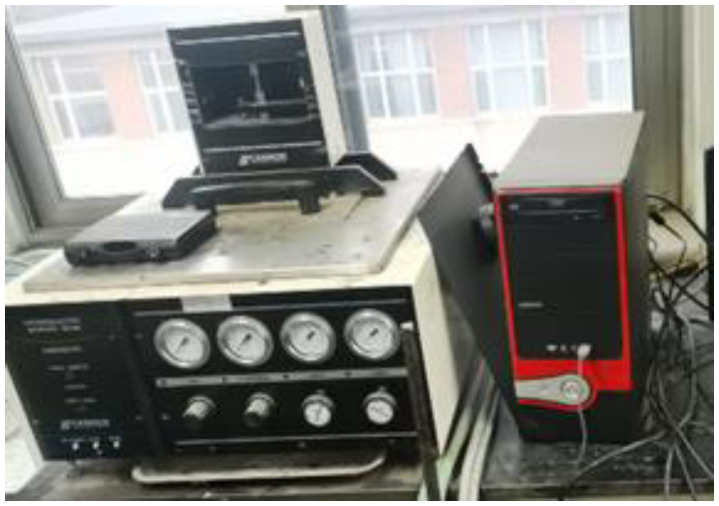
The bending beam rheometer.

(a) Temperature sweep test: The temperature sweep test was conducted in accordance with the specification requirements of JTG E20-2011. The test adopted the strain control mode, and the strain value was 12%. The temperature range was 58~88 °C with an interval of 6 °C. Then, the high temperature performance was evaluated using complex modulus (G*), phase angle (δ), and rutting factor (G*/sinδ).

(b) MSCR test: The MSCR test was conducted in accordance with the specification requirements of AASHTO T350-14 [22]. The test temperature was 64 °C, and the stress levels were 0.1 kPa and 3.2 kPa. After the test, the rutting resistance was evaluated using creep recovery rate (R) and non-recoverable creep compliance (Jnr).

(c) LAS test: The MSCR test was conducted in accordance with the specification requirements of AASHTO TP 101-14 [23], The test temperature was 25 °C, and the fatigue performance was characterized by integrity parameter (C), damage factor (D(t)), and fatigue life (N_f_). It is worth noting that the temperature sweep test, MSCR test, and LAS test are all performed using a dynamic shear rheometer (KNX2110) manufactured by Malvern Instruments Limited in Shanghai, China. The dynamic shear rheometer is shown in Figure 1.

(d) BBR test: The MSCR test was conducted in accordance with the specification requirements of JTG E20-2011. The test temperature was −6 °C, −12 °C, and −18 °C, and the low temperature performance was evaluated using creep stiffness (S) and creep rate (m). Among them, the BBR test is conducted using a bending beam rheometer (TE-BBR-F) manufactured by Cannon Instrument Company in Shanghai, China, as shown in Figure 2.

#### 2.2.5. FTIR Test

**Figure 3:**
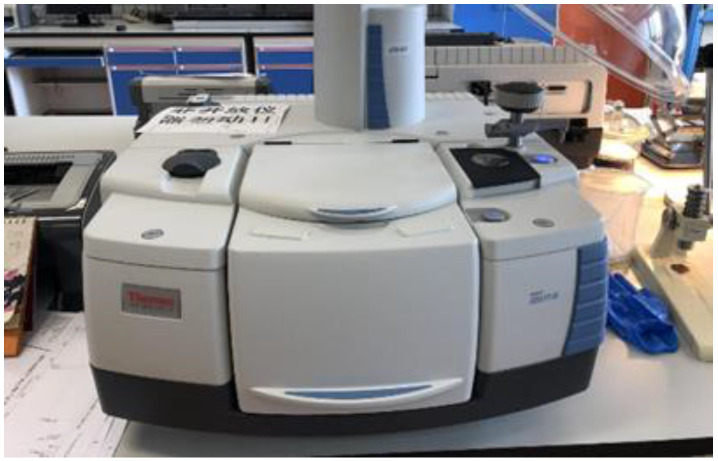
The FTIR test device.

To characterize the modification mechanism of high-modulus asphalt, a spectrometer was used to analyze the chemical structure of the asphalt samples. The test range was 4000~400 cm^−1^ with a resolution of 4 cm^−1^. In addition, the FTIR test was performed using a device (IS50) manufactured by Thermo Nicolet in Waltham, MA, USA, which is shown in Figure 3.

## 3. Results

### 3.1. Development of Modified High-Modulus Asphalt

Compared with ordinary asphalt, high-modulus asphalt should meet the index requirement of G* ≥ 10 kPa, and its physical properties should also comply with the specification of DB 37/T 3564—2019 [24]. Therefore, penetration, softening point, ductility, and G* were used as evaluation indicators, and orthogonal tests were conducted on the formula of modified high-modulus asphalt according to Table 3 [25], with the results shown in Table 4.

Table 4 presents the test results of the orthogonal test and the calculation results of intuitive analysis. Compared with VA, the penetration and ductility of several modified asphalts generally show attenuation in varying degrees, indicating that the incorporation of modifiers increases the compactness of asphalt and weakens its flow performance. Additionally, the softening points and G* of these modified asphalts significantly increase with the addition of modifiers, suggesting that modifiers effectively enhance the high temperature performance and deformation resistance of virgin asphalt. This is because the modifiers, mainly powdery materials, fill the pore structure with asphalt during mixing, improving its compactness and thus endowing it with remarkable high-temperature deformation resistance. Although penetration and ductility can characterize the low temperature performance of asphalt to some extent, their attenuation does not imply gradual deterioration of low temperature performance, which will be further explored through the BBR test in the subsequent section.

Interestingly, intuitive analysis of several performance indicators shows that A_3_ and B_3_ are generally the optimal dosages for WR and EVA, while the recommended dosage of PPA is less obvious. Therefore, this study conducted experimental analyses on modified asphalts with three mix ratios (A_3_B_3_C_1_, A_3_B_3_C_2_, and A_3_B_3_C_3_), and the results are shown in Table 5. It can be seen in Table 5 that increasing the PPA dosage first increases and then decreases the penetration and G*, while the ductility increases but the softening point gradually decreases. Comprehensive analysis shows that the modified high-modulus asphalt achieves optimal test results when the mix ratio of modifiers is A_3_B_3_C_2_. Therefore, based on the orthogonal test and relevant exploratory test results, this study recommends the formula of modified high-modulus agent as 20% WR, 6% EVA, and 4% PPA. The self-developed modified high-modulus asphalt is named WRA.

### 3.2. Rheological Tests

#### 3.2.1. Temperature Sweep Test

**Figure 4:**
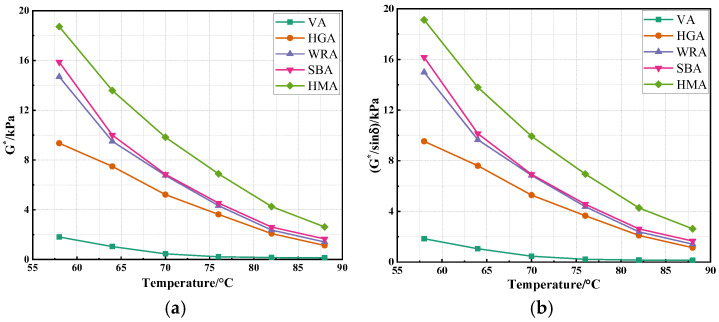
The results of temperature sweep test: (**a**) G*; (**b**) G*/sinδ.

This study explored the variation trend of high temperature performance of asphalt through a temperature sweep test and further evaluated the high temperature performance of several high-modulus asphalts [26]. The results are shown in Figure 4.

As shown in Figure 4, the variation trend of G*/sinδ is similar to G*. At the same temperature, the order of G*/sinδ and G* for high-modulus asphalts is HMA > SBA > DPA > HGA, indicating that high-modulus asphalts prepared with high-modulus agents and SBS modifiers exhibit the best high temperature performance, followed by WRA and HGA. This is because high-modulus agents and SBS modifiers are high-molecular polymers that form a stable three-dimensional network structure in asphalt after blending, enhancing its structural stability, reducing fluidity, and improving high temperature deformation resistance. Although WR, synergistically with PPA and EVA, can effectively reduce asphalt fluidity and increase viscosity, it lacks the properties to form a three-dimensional network, resulting in a relatively lower high temperature performance of WRA. Notably, while the high temperature performance of WRA is slightly inferior to HMA and SBA, its G* value at 60 °C far exceeds 10 kPa, confirming that WRA meets the application requirements for high-modulus asphalt. Additionally, as the temperature increases, both G*/sinδ and G* of the high-modulus asphalts gradually decrease, indicating that elevated temperatures adversely affect their high temperature performance. This is because increased temperatures soften the asphalt, enhance its fluidity, and thus reduce deformation resistance. Interestingly, the performance grade (PG) of all high-modulus asphalts is PG 88, demonstrating their fitness for service in high temperature environments in practical engineering.

#### 3.2.2. MSCR Test

**Figure 5:**
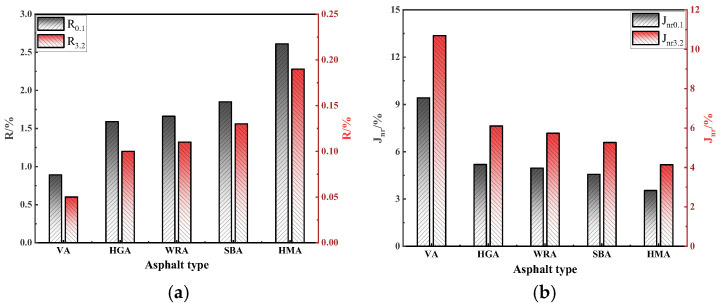
The results of the MSCR test: (**a**) R; (**b**) Jnr.

The MSCR test can characterize the rutting resistance of asphalt, further evaluating its deformation resistance at high temperatures. Therefore, this study conducted the MSCR test on several high-modulus asphalts and evaluated their rutting resistance using R and Jnr under different strain levels [27]. The results are shown in Figure 5.

As shown in Figure 5, under different stress levels, the R and Jnr of several high-modulus asphalts exhibit similar patterns, as the stress level increases, R gradually decreases while Jnr increases. R and Jnr characterize the rutting resistance of asphalt, the higher the R and the lower the Jnr, the better the rutting resistance, which indicates that an increase in stress level deteriorates the rutting resistance of asphalt. This is because excessive stress accelerates the deformation process of asphalt, leading to a decline in rutting resistance. Consistent with the temperature sweep test results, the rutting resistance of high-modulus asphalts ranks as HMA > SBA > WRA > HGA. High-modulus asphalts prepared with two polymer modifiers show better rutting resistance than WRA, as polymer modifiers form a stable three-dimensional network structure in asphalt after blending, enhancing its structural stability and resistance to loading. Notably, although the rutting resistance of WRA is lower than HMA, it is nearly comparable to SBA. This suggests that under the synergistic effect of PPA and EVA, WR can effectively enhance the rutting resistance of asphalt. Although WR cannot form a network structure, it absorbs light components in asphalt to adjust the viscoelastic ratio, while PPA and EVA form cross-linking structures with asphalt to further improve high temperature deformation resistance. Overall, despite a slight gap with polymer-based high-modulus asphalts, WRA demonstrates remarkable rutting resistance.

#### 3.2.3. LAS Test

**Figure 6:**
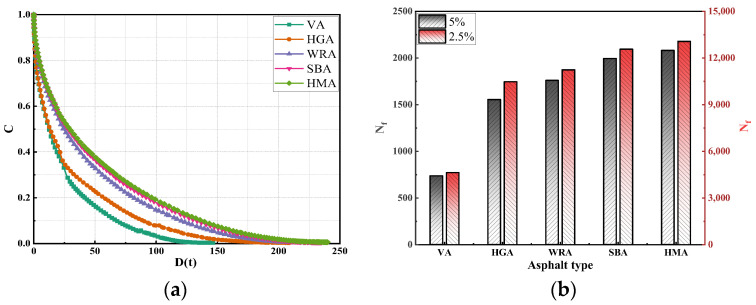
The results of LAS test: (**a**) C; (**b**) N_f._

During the service life, asphalt pavements should possess sufficient fatigue resistance to withstand repeated vehicle loads, especially high-modulus asphalt, which has a higher elastic modulus and deformation resistance and should exhibit more remarkable fatigue performance. To evaluate the fatigue performance of WR, this study conducted a LAS test on several high-modulus asphalts [28], and evaluated their fatigue performance using C, D(t), and N_f_. The results are shown in Figure 6.

As seen in Figure 6, this study calculated the fatigue performance indices of several high-modulus asphalts, where C and D(t) characterize the integrity of the asphalt. When the C value is 1 and the D(t) value is 0, the asphalt is in a complete state; when the C value is 0, the asphalt is completely damaged, and the D(t) value currently also reflects the asphalt’s resistance to fatigue damage to a certain extent. It can be observed that the variation order of D(t) for several asphalts is HMA > SBA > DPA > HGA, indicating that polymer modifiers have a more significant enhancement effect on the fatigue performance of asphalt. The reason is similar to the previous rheological test results: the network structure formed by polymers inside the asphalt effectively enhances the structural stability of the asphalt, making it less prone to shear failure under external forces. Although DRP reduces the fluidity of asphalt by filling the internal voids and absorbing some light components, it does not essentially change the structural characteristics of the asphalt. The cross-linking effect of PPA and EVA only further increases the viscosity of the asphalt, making it more in line with the functional characteristics of high-modulus asphalt. The synergistic effect of the three does not change the structural characteristics of the asphalt, thus the D(t) of WRA is lower than high-modulus asphalts prepared with the two polymer modifiers.

Notably, the D(t) of WRA is significantly higher than HGA, indicating that although the fatigue performance of WRA has not reached the level of polymer-modified asphalt, it still far exceeds that of hard asphalt. This confirms that the high-modulus asphalt developed using WR as the main material meets the fatigue performance requirements. Interestingly, the analysis of D(t) is validated by the N_f_. To evaluate the fatigue life of high-modulus asphalts, N_f_ under different strain conditions (5% and 2.5%) was calculated, showing that N_f_ decreases with increasing strain. This indicates that higher loads exacerbate the degradation of asphalt fatigue performance. Additionally, the order of N_f_ for the asphalts is HMA > SBA > DPA > HGA, further verifying the D(t) analysis results.

#### 3.2.4. BBR Test

**Figure 7:**
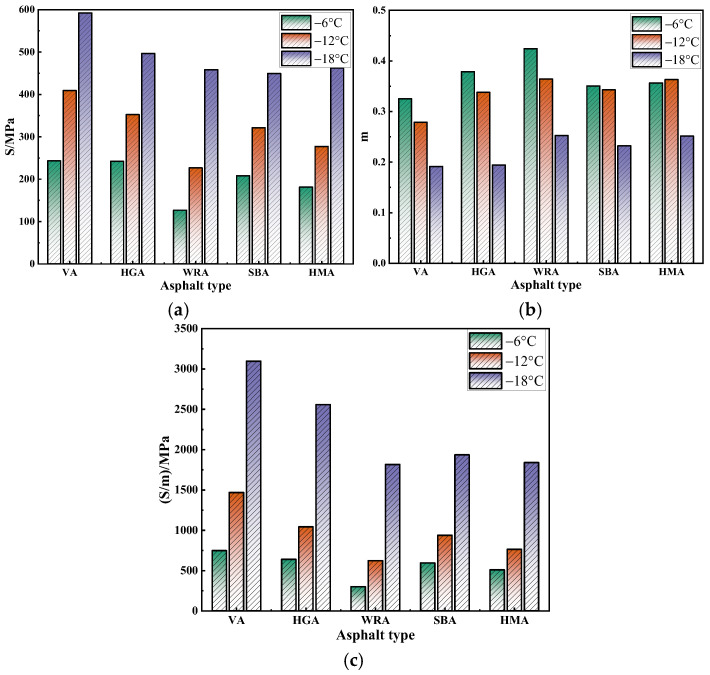
The results of the BBR test: (**a**) S; (**b**) m; (**c**) S/m.

Low-temperature performance is one of the key factors affecting the service capacity of asphalt pavements. To characterize the low temperature performance of several high-modulus asphalts, this study conducted a BBR test [29,30] and evaluated their low temperature performance using S and m. The results are shown in Figure 7.

As shown in Figure 7, the variation patterns of S and m for several asphalts are opposite, as the temperature decreases, S gradually increases while m decreases. A higher S value and lower m value indicate poorer low temperature performance, suggesting that decreasing temperature adversely affects the low temperature performance of asphalt. Additionally, different asphalt types lead to variations in low temperature performance. Notably, WRA exhibits the lowest S value and highest m value at all test temperatures, indicating that its low temperature performance significantly surpasses that of asphalts modified with a high-modulus agent or SBS. This differs from the results of other rheological tests, primarily because WR fills the voids in asphalt, enhancing the interlocking and anchoring effect between the asphalt and WR. Moreover, the high elasticity of WR promotes the elastic behavior of asphalt, while the synergistic effect of PPA and EVA further strengthens the cross-linking between WR and asphalt, thereby improving the cracking resistance at low temperatures. Interestingly, at the same temperature, the order of S values for the asphalts is WRA < HMA < SBA < HGA, while the order of m values is WRA > HGA > HMA > SBA. The discrepancy in these orders indicates that evaluating low temperature performance solely based on S or m values may introduce certain errors. Based on relevant research experience [31], this study further evaluated the low temperature performance of several asphalts using the S/m value. As shown in Figure 7c, the variation pattern of S/m values is highly consistent with S values, the lower the S/m value, the better the low temperature performance. The order of S/m values for the asphalts is WRA < HMA < SBA < HGA, indicating that WRA exhibits the most significant low temperature performance, followed by HMA, SBA, and HGA. Notably, the S/m values of HGA at different temperatures are significantly higher than other asphalts, which aligns with previous findings that hard asphalt has poor low temperature performance. Furthermore, to characterize the low temperature performance grades of the asphalts, the study referred to the standard of AASHTO M320 [32] (m ≥ 0.3; S ≤ 3 00 MPa). The results showed that the low temperature limit temperature of WRA and HMA is −12 °C, while that of SBA and HGA is −6 °C. This further confirms that the modified high-modulus asphalt developed in this study possesses remarkable low temperature performance.

### 3.3. FTIR Test

**Figure 8:**
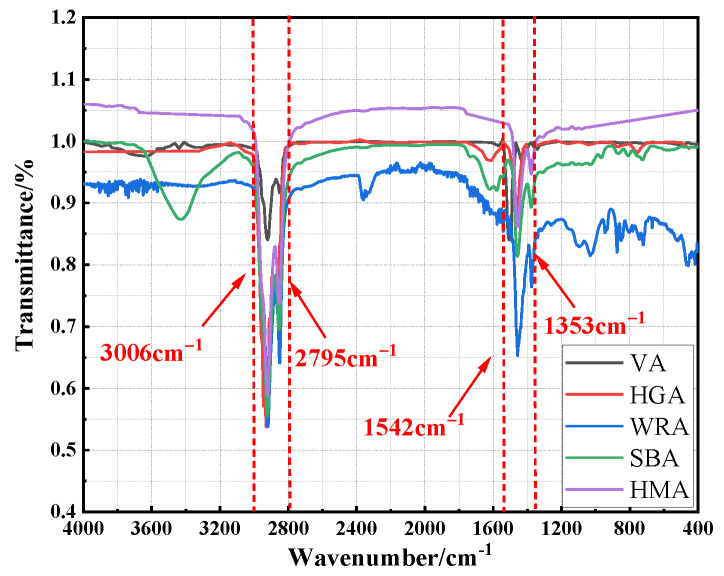
The FTIR test result of several asphalts.

The variation in chemical structure of asphalt significantly influences the stability of its service performance. Differences in modifiers among various high-modulus asphalts will inevitably lead to variations in the reaction types between modifiers and asphalt, thereby affecting the stability of the interface structure layer between asphalt and modifiers in the asphalt system. To further evaluate the modification mechanism of WRA, this study conducted a FTIR test on several high-modulus asphalts [33], and the results are shown in Figure 8.

As seen in Figure 8, several types of asphalt exhibit distinct characteristic peaks at 2795~3006 cm^−1^ and 1353~1542 cm^−1^, which are generally caused by the stretching vibration and out-of-plane bending vibration of C-H bonds, characterizing the asphaltene content inside the asphalt. Compared with the VA, the characteristic peak intensities of several high-modulus asphalts in this place are significantly increased, meaning that the proportion of asphaltene components inside several high-modulus asphalts greatly increased. This is because HGA is hard asphalt, which usually contains a low concentration of light components, thus the proportion of its asphaltene components is extremely high. For WRA, SBA, and HMA, it is because the modified materials absorb some light components inside the asphalt during the preparation process, resulting in an increase in the proportion of heavy components and finally causing the change in the peak intensity of the characteristic peaks. Additionally, although the peak intensity of HMA’s characteristic peaks changed, no new characteristic peaks emerged, indicating that the high-modulus modifier only physically mixed with the asphalt during modified asphalt preparation without obvious chemical reactions. Both WRA and SBA exhibited subtle characteristic peaks in the 400~1200 cm^−1^ band, but the peak fluctuations of SBA in this band were extremely weak, likely due to the response of the benzene ring structure in SBS during its mixing with asphalt. By contrast, WRA showed remarkable peak fluctuations in the 400~1200 cm^−1^ band, during the preparation process of WRA, PPA in the modifier undergoes an esterification reaction with the -OH groups in asphalt, increasing the content of macromolecules in the asphalt, enhancing its viscosity, and ultimately improving its elastic behavior under stress, which suggests that the modification process of WRA is a chemical modification.

### 3.4. Fluorescence Microscopy FM Test

**Figure 9:**
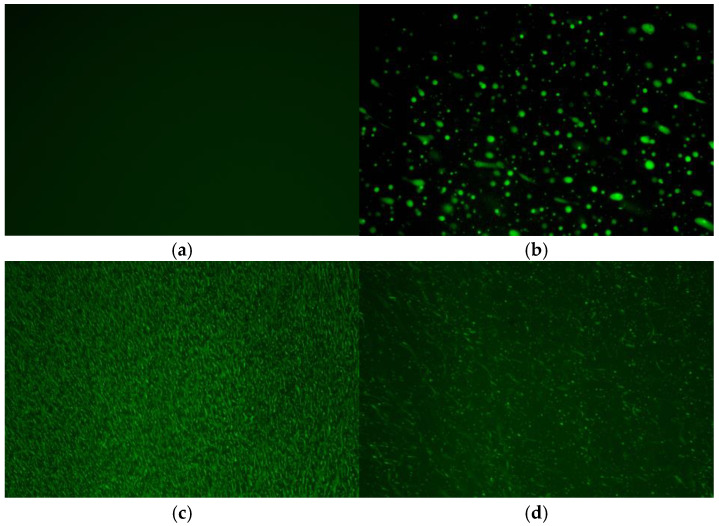
The test result of several asphalts: (**a**) HGA; (**b**)WRA; (**c**) SBA; (**d**) HMA.

The preparation of high-modulus asphalt mainly relies on modification materials such as polymers and thermoplastic elastomers. When these materials are mixed with asphalt, swelling often occurs. The swelling degree of modification materials in asphalt significantly affects the structural stability and service performance of modified asphalt. Therefore, this study characterized the microstructure of several asphalts through a fluorescence microscopy test to analyze the swelling properties of different modification materials [34,35]. The results are shown in Figure 9.

As seen in Figure 9, the microstructures of several high-modulus asphalts differ significantly. The microstructure of HGA only presents a dark cyan asphalt phase, because HGA is hard asphalt without any modified materials inside, thus the green modified material phase does not appear. The boundaries between the asphalt phase and the polymer phase in SBA and HMA are not significant. Compared with HGA, SBA and HMA are filled with polymer phases, which are uniformly distributed inside the asphalt to form a relatively stable network structure, thereby enhancing the structural stability of the asphalt. Additionally, some molecular chains in the polymer modifiers diffuse into the asphalt during mixing, adsorbing light components and enhancing the elastic behavior of asphalt to a certain extent, which explains the superior rheological properties of SBA and HMA. Although WRA also shows many modified material phases, it does not form a network structure like SBA and HMA, indicating that the swelling effect of WR in asphalt is lower than that of polymer modifiers. This is because the swelling of WR particles in asphalt is affected by adjacent WR particles, leading to relatively weak structural stability, which further explains the reason that the rheological properties of WRA are slightly inferior to SBA and HMA.

## 4. Conclusions

This study prepared a new high-modulus asphalt with WR through modification technology and characterized the physical and rheological properties of several high-modulus asphalts via conventional performance tests and rheological tests. The main conclusions are as follows:

(1) This study analyzed the formula of high modulus asphalt modified by WR through orthogonal tests and determined the recommended blending ratio as 20%WR, 6% EVA, and 4% PPA.
(2) The high temperature grade of WRA and other high-modulus asphalts is PG 88, indicating that WRA has excellent high temperature deformation resistance. Although its high temperature performance, rutting resistance, and fatigue performance are slightly lower than those of SBA and HMA, they are significantly higher than HGA, which is caused by differences in the internal structure of asphalt.
(3) WRA exhibits significantly better low temperature performance than other asphalts, which is because WR fills the voids in asphalt, enhancing the interlocking and anchoring effect between asphalt and WR. The synergistic effect of PPA and EVA further strengthens the cross-linking between WR and asphalt, thereby improving the cracking resistance at low temperatures.
(4) The infrared spectrum of WRA shows new characteristic peak fluctuations, indicating that the modification process of WRA is chemical modification. In addition, compared with VA, the peak intensities of several high-modulus asphalts at 2795.34~3006.06 cm^−1^ and 1353.91~1542.03 cm^−1^ are significantly enhanced, suggesting that the modification process reduces the proportion of light components in asphalt. Compared with SBA and HMA, WRA has a lower swelling effect in asphalt and does not form an obvious network structure.

## Figures and Tables

**Table 1:** Basic properties of asphalt.

Properties	Index	VA	HGA	HMA	SBA
Penetration	(25 °C)/0.1 mm	71.5	24.2	28.5	33.6
Ductility	(10 °C)/cm	24.1	20.1	22.3	32.5
Softening point	°C	46.7	70.9	71.6	77.2
RTFOT (163 °C, 85 min)	Mass loss rate/%	−0.55	−0.45	−0.44	−0.41
	Penetration ratio/%	72	76	75	73
	Residual ductility/cm	7.1	8.4	11.6	14.7

**Table 2:** Basic properties of modified materials.

Material	Indexes	Values
Waste rubber powder (WR)	Metal content, %	0.02
	Ash content, %	7.1
	Carbon content, %	3
	Rubber hydrocarbon, %	54
	Exterior	Black powder
Ammonium polyphosphate (PPA)	Water content, %	0.12
	Density, g/cm^3^	1.85
	Thermal decomposition temperature, °C	289
	Exterior	White powder
Ethylene-vinyl acetate copolymer (EVA)	Density, g/cm^3^	0.945
	Melting point, °C	103
	Thermal decomposition temperature, °C	241
	Exterior	Transparent particle

**Table 3:** Factor level table.

Level	(A) DRP/%	(B) EVA/%	(C) PPA/%
1	10	4	2
2	15	5	3
3	20	6	4

**Table 4:** The results of the orthogonal test.

Number	Factor			Evaluation Indexes			
	A	B	C	Penetration/(25 °C/0.1 mm)	Softening point/°C	Ductility/(10 °C/cm)	G*/(60 °C)
1	1	1	1	31.3	70.3	16.3	7.4
2	1	2	3	30.5	69.7	17.2	8.1
3	1	3	2	28.2	70.5	18.6	9.2
4	2	1	3	28.4	70.2	18.4	10.1
5	2	2	2	27.1	70.6	19.1	10.7
6	2	3	1	27.8	71	19.7	11.3
7	3	1	2	26.2	70.2	22.3	12
8	3	2	1	25.6	71.2	23.4	12.4
9	3	3	3	25.1	70.7	25.3	12.5
K1	30.0	28.6	28.2	Intuitive analysis of penetration			
K2	27.8	27.7	27.2				
K3	25.6	27.0	28.0				
R	4.4	1.6	1.1				
Solution	A_1_	B_1_	C_1_				
K1	70.2	70.2	70.8	Intuitive analysis of softening point			
K2	70.6	70.5	70.4				
K3	70.7	70.7	70.2				
R	0.5	0.5	0.6				
Solution	A_3_	B_3_	C_1_				
K1	17.4	19.0	19.8	Intuitive analysis of ductility			
K2	19.1	19.9	20.0				
K3	23.7	21.2	20.3				
R	6.3	2.2	0.5				
Solution	A_3_	B_3_	C_3_				
K1	8.2	9.8	10.4	Intuitive analysis of G*			
K2	10.7	10.4	10.6				
K3	12.3	11.0	10.2				
R	4.1	1.2	0.4				
Solution	A_3_	B_3_	C_2_				

**Table 5:** The test results of several asphalts.

Properties	Index	A_3_B_3_C_1_	A_3_B_3_C_2_	A_3_B_3_C_3_
Penetration	(25 °C)/0.1 mm	26.3	27.2	26.2
Ductility	(10 °C)/cm	24.4	25.1	25.9
Softening point	°C	71.5	71.2	70.9
G*	kPa	11.8	13.3	12.8

## Data Availability

The original contributions presented in this study are included in the article. Further inquiries can be directed to the corresponding authors.
